# Recent Progress in Inkjet‐Printed Thin‐Film Transistors

**DOI:** 10.1002/advs.201801445

**Published:** 2019-01-11

**Authors:** Seungjun Chung, Kyungjune Cho, Takhee Lee

**Affiliations:** ^1^ Photo‐Electronic Hybrids Research Center Korea Institute of Science and Technology Hwarang‐ro 14‐gil 5 Seongbuk‐gu Seoul 02792 South Korea; ^2^ Department of Physics and Astronomy, and Institute of Applied Physics Seoul National University Seoul 08826 South Korea

**Keywords:** flexible devices, inkjet printing, solution processes, switching devices, thin‐film transistors

## Abstract

Drop‐on‐demand inkjet printing is one of the most attractive techniques from a manufacturing perspective due to the possibility of fabrication from a digital layout at ambient conditions, thus leading to great opportunities for the realization of low‐cost and flexible thin‐film devices. Over the past decades, a variety of inkjet‐printed applications including thin‐film transistors (TFTs), radio‐frequency identification devices, sensors, and displays have been explored. In particular, many research groups have made great efforts to realize high‐performance TFTs, for application as potential driving components of ubiquitous wearable electronics. Although there are still challenges to enable the commercialization of printed TFTs beyond laboratory‐scale applications, the field of printed TFTs still attracts significant attention, with remarkable developments in soluble materials and printing methodology. Here, recent progress in printing‐based TFTs is presented from materials to applications. Significant efforts to improve the electrical performance and device‐yield of printed TFTs to match those of counterparts fabricated using conventional deposition or photolithography methods are highlighted. Moreover, emerging low‐dimension printable semiconductors, including carbon nanotubes and transition metal dichalcogenides as well as mature semiconductors, and new‐concept printed switching devices, are also discussed.

## 1 Introduction

Next‐generation electronics, also called the Internet of Things (IoT), will closely interface with our physical worlds via a variety of sensors, communication devices, and displays.^1^, ^2^, ^3^, ^4^, ^5^ As the demands for wearable or implantable devices/systems increase, new solutions in terms of suitable materials and processes are highly desirable. Since the 1960s, the microelectronics market has dramatically advanced with the semiconductor industry, which requires complex semiconductor processing on silicon‐based platforms.^6^, ^7^, ^8^ However, future electronics may need to be free from rigid substrates fabricated through a series of subtractive processes, such as traditional evaporation processes with masks or photolithography followed by etching. In other words, devices should be implemented on substrates other than limited rigid silicon or glass, and miniaturized even at additional cost. In particular, the ability to realize flexible thin‐film transistors (TFTs), which are key driving/switching component of wearable electronics, offers much freedom on the target substrates. Therefore, a variety of functional materials focusing on semiconductors have been extensively explored for realizing competitive flexible TFTs, including traditional silicon,^9^, ^10^, ^11^ organics,^12^, ^13^, ^14^, ^15^, ^16^, ^17^ oxides,^18^, ^19^, ^20^, ^21^ carbon nanotubes (CNTs),^22^, ^23^, ^24^, ^25^, ^26^ and emerging 2D materials.^27^, ^28^, ^29^, ^30^ Furthermore, because representative flexible or stretchable platforms, such as polymer‐based plastic and polydimethylsiloxane (PDMS) substrates, are difficult to utilize in traditional microfabrication, the development of alternative processes that can be employed for implementing low‐cost, large‐area, flexible, and biocompatible electronics is key to meet this demand.

**Figure 1:**
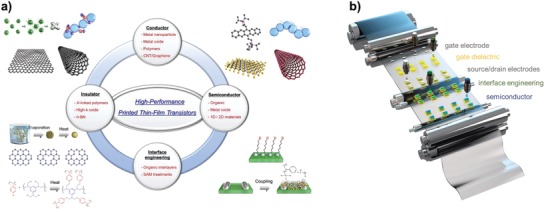
a) Functional electronic inks for realizing high‐performance inkjet‐printed TFTs. b) Scheme of sequential inkjet printing process on a flexible substrate.

Additive printing is one of the most promising candidates to satisfy these requirements and is a well‐suited strategy to implement commercial thin‐film devices and systems because of its large‐area, ultra low‐cost, nonvacuum, and environmentally friendly processability.^31^, ^32^, ^33^, ^34^, ^35^, ^36^ This attractive approach was originally employed in the graphic art industry to make patterns on ink‐wettable platforms, such as fabric and papers. As a result, it has a prominent position in mass production lines. Sophisticated printing machines and functional inks have been gradually developed over hundreds of years, and thus printed patterns can be produced on very‐large‐area targets that are several meters wide at high‐printing‐speeds on the order of 10 m s^−1^. These achievements have facilitated large‐production throughput, and enabled low‐cost production per unit area. Printed electronics can leverage the high‐throughput additive manufacturing process demonstrated at low‐cost in graphic arts applications.^37^, ^38^, ^39^ Here, the additive nature allows fast processing and imparts cost savings by depositing the materials where they ultimately need to be located. Thus, minimization of both material waste and process steps for fabrication is possible, in contrast to subtractive semiconductor processing.^40^, ^41^, ^42^ Another key advantage is that printed features are manufactured via solution‐processes, so that flexible electronics can be facilely implemented on large‐area substrates without using any vacuum‐assisted deposition methods and by low‐temperature processing methods when appropriate ink solvents are used. In this regard, over the past decades, a wide range of flexible thin‐film devices, including transistors, light‐emitting devices, sensors, micro‐electromechanical systems (MEMs), energy harvesting and storage devices, and radio‐frequency identification (RFID) antennas, have been fabricated using printing techniques.^43^, ^44^, ^45^, ^46^, ^47^, ^48^, ^49^, ^50^ In particular, additive printing has great advantages for realizing stack‐structured TFTs consisting of conductive, insulation, and semiconductor layers on flexible substrates with a low thermal budget, even below 200 °C when organic or nanoparticle‐type functional inks are used (**Figure** **1**).

**Figure 2:**
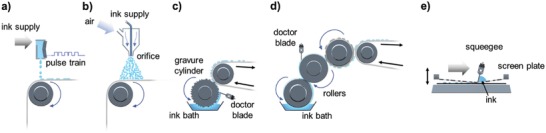
Schemes of various printing techniques: a) inkjet printing, b) spray printing, c) gravure printing, d) flexographic printing, and e) screen printing.

The printing techniques can be roughly classified into nozzle‐based digital inkjet printing and nondigital screen, offset, flexography, and gravure printing, as shown in **Figure** **2**. Because of the relatively poor resolution and complex processing in screen and flexography printing, respectively,^51^, ^52^ gravure and inkjet printing among the different printing techniques have been widely used as suitable processing methods for the realization of thin‐film devices.^53^, ^54^, ^55^ Specifically, gravure printing allows high‐resolution, high‐throughput, and good pattern fidelity for fabrication.^37^, ^38^, ^39^, ^56^, ^57^, ^58^ However, the inherent contact nature of gravure printing and the use of high‐viscosity ink with binders cause contamination/residue issues and degradation of the printing materials, respectively. In addition, gravure printing has lower registration accuracy for vertically stacked printed layers.^58^, ^59^ Although many advanced results for gravure‐printed TFTs have been reported by pushing into the highly scaled regime to produce a printed line width of ≈2 µm at high‐speeds on the order of 1 m s^−1^,^59^, ^60^, ^61^, ^62^ it is still difficult to determine the best candidate for complicated stacked devices. Inkjet printing has lower resolution and slower deposition speed than its counterpart of gravure printing, which is fully determined by the diameter of the nozzles and speed of the motion stage and printer head, respectively. Moreover, there is a trade‐off between scaling and throughput limited by the need to mechanically raster a print head across large substrates. However, the noncontact nature of inkjet printing and the use of binder‐less inks can realize high‐quality printed features without unwanted residual patterns or additional rinsing processes. Moreover, inkjet printing has higher registration accuracy, which allows the fabrication of devices with complex stacked structures without masks.^63^ Finally, because inkjet printing is a type of drop‐on‐demand (DOD) digital printing, physically pre‐encoded patterns in rollers or plates are not required; therefore, customized production and short print‐runs with rapid design changes can be achieved with much freedom.^64^, ^65^, ^66^ Previous studies on printed TFTs have predominantly focused on an inkjet printing methodology, which utilizes piezoelectric DOD jetting of picoliter (pL)‐volume droplets.^54^, ^67^, ^68^, ^69^, ^70^

This review highlights recent progress in inkjet‐printed TFTs to achieve improved electrical performance and their emerging electronics applications. Although several excellent reviews have already introduced printable organic, oxide, and 2D materials for large‐area TFT applications, for example, high‐speed printed circuits^41^ and transparent CNT‐based applications,^25^ the overarching aim of this review is to provide an overview of the recent efforts to realize high‐performance inkjet‐printed TFTs based on various channel materials and switching mechanisms as well as fundamental inkjet printing technology. The issues raised in the field of inkjet‐printed organic, metal‐oxide, low‐dimension thin‐film transistors in electronic and material perspectives would be systematically described, and then the currently promising strategies to address these issues will be introduced. In Section 2, we briefly introduce the background of inkjet printing focusing on a piezoelectric‐type inkjet printing system. To provide a thorough review and comparison, another printing candidate is also briefly discussed. Then, emerging functional semiconductor inks, especially those compatible with flexible substrates, are introduced. In Section 3, recent achievements in inkjet‐printed TFTs with organic, metal oxide, CNT, and 2D channels are discussed. Additionally, emerging printed mechanical switching devices, i.e., MEM relays, that can address fundamental limitations in the energy efficiency of traditional field‐effect transistors by minimizing the off current and subthreshold swing (SS) values are introduced. Therefore, we believe this review article can be beneficial for the readers by reporting 1) the fundamental inkjet printing, 2) the state‐of‐art of inkjet‐printed TFTs, 3) current issues in various inkjet‐printed TFTs depending on semiconductor materials and the efforts to address them, and 4) new‐type of inkjet‐printed switching devices.

## 2 Fundamental Description of Inkjet Printing and Electronic Materials

### 2.1 Background of Inkjet Printing Technology

Inkjet printing is one of the most effective printing technologies for enabling additive manufacturing using soluble materials. This methodology has achieved significant progress in the fabrication of thin‐film electronics. This section will introduce basic principles of inkjet printing for thin‐film formation.

#### 2.1.1 Inkjet Printing System

Inkjet printers consist of basic three parts: the motion stage, the vision system, and the control systems including the print heads. The nozzle heads are directly connected to a reservoir or cartridge containing the ink, and the pressure control system applies pneumatic force (pulse train) not only to push the ink to the head, but also to define the ink meniscus at the nozzle orifice.

**Figure 3:**
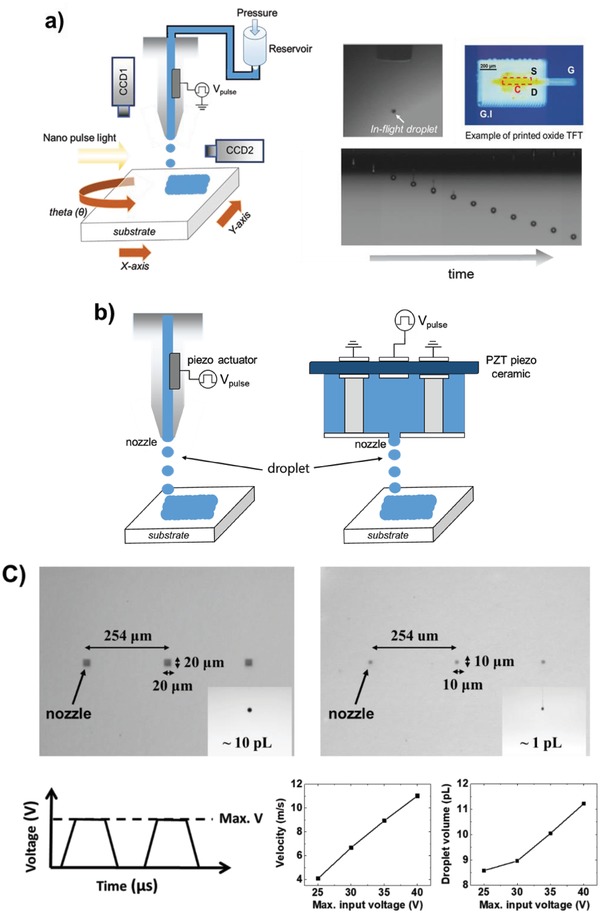
a) Inkjet printing system, captured image of an in‐flight ink droplet from (top) a single nozzle, (bottom) cartridge‐type nozzle, and example optical image of a printed TFT (G: gate, S and D: source and drain electrodes, C: channel). b) Schematic of droplet ejection in piezoelectric nozzles: (left) a single nozzle, (right) cartridge‐type nozzle. c) Top‐view optical images of the cartridge‐type nozzles having a diameter of 20 µm (left) and 10 µm (right). (The ejected droplets having a volume of ≈10 pL (left) and ≈1 pL (right) in flight are also included.) The droplet size and velocity from a 20 µm diameter nozzle depending on the maximum voltage of the input pulse train.

Inkjet printing systems typically require three mechanical degrees of freedom, two translational (X and Y stages) and one rotational (Θ stage) to create 2D patterns and align with previously printed patterns to realize facilely stacked structures, respectively. A vision system consisting of at least two cameras is also necessary to enable substrate alignment (fiducial camera) and to observe ejected droplets in flight (drop‐watching camera) to obtain well‐defined droplets. Control systems are employed to optimize the temperature of both the stage and the nozzle, which can directly affect the substrate temperature and the dropping velocity, respectively (scheme of a piezoelectric type inkjet‐printer is shown in **Figure** **3**a).

#### 2.1.2 Ink Droplet Formation

The ejected droplet formation mechanism is largely classified into two systems using 1) thermal nozzles and 2) piezoelectric nozzles.^70^ Thermal nozzles employ a heater in the form of a resistor. If the internal temperature is enough to create a bubble in a reservoir by increasing current, it facilitates the single droplet formation of ink out of the nozzle via volume expansion. Then, the negative pressure, forming inside the reservoir after the droplet has been ejected, draws new ink inside the reservoir. To utilize thermal nozzles to create droplets, the inks must be heat‐compatible and sensitive to the volume contraction/expansion depending on the temperature. By contrast, piezoelectric nozzles contain a piezoelectric film placed along the wall of a reservoir, so the deformation of the film drives the mechanical volume expansion by applying voltage pulses. Then, the ink can be ejected in response to the pressure generated by the piezoelectric element (Figure 3b). Therefore, piezoelectric nozzles with relatively better resolution, requiring lower temperature, and enabling more precise operation are preferred for realizing printed devices that do not suffer from ink degradation concerns and temperature‐sensitive solvent choice, although thermal nozzles are typically less expensive and widely used in commercial printers. Note that the droplet size and velocity in flight are dominantly determined by the diameters of nozzles and maximum voltage of the input pulse train (Figure 3c). Therefore, we will describe inkjet printing focusing on a piezoelectric type in this review.

Stable drop formation without satellite droplets after ejection from the nozzles is also important to obtain well‐defined printed patterns on a substrate. This behavior closely incorporates the transfer of kinetic energy from the nozzles to the ejected droplets. In the initial state, the ink in the nozzle is in equilibrium. Upon applying a voltage pulse signal into the piezoelectric element, the ink extrudes out due to the volume expansion inside the nozzle. After kinetic energy over a threshold is transferred to the extruded ink, an in‐flight droplet is generated and then drops toward the target substrate. In the sequentially applied opposite voltage, the ink in the nozzles refills, and the process repeats. As mentioned before, in DOD inkjet printing, a droplet is generated by separation from the ink inside the nozzle when the piezoelectric element is actuated. This behavior means that an idle state, i.e., nonjetting condition, applies a small voltage pulse to the nozzle during the nonjetting condition to avoid ink drying and nozzle clogging.

**Figure 4:**
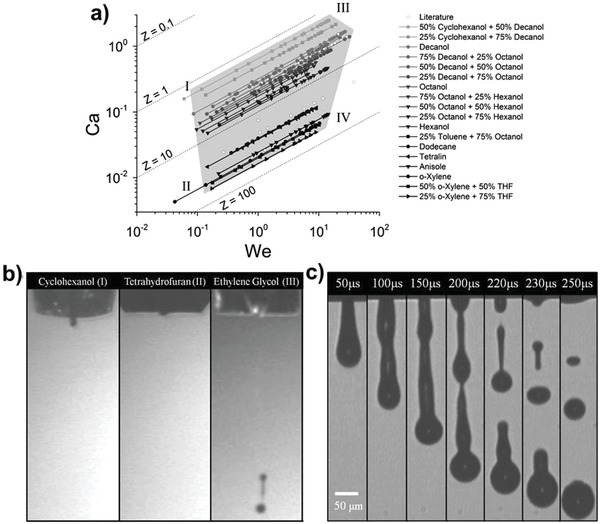
a) Jettable window within the capillary number–Weber number parameter space. The solvent systems used to create the window are oxylene, tetralin, anisole, decanol, hexanol, octanol, and binary solvent mixtures of hexanol/octanol, octanol/decanol, decanol/cyclohexanol, and oxylene/tetrahydrofuran. b) Drop stability breakdown mechanisms corresponding to the first four regions of the jettability window. c) Multiple droplet breakups resulting from wavelike instability corresponding to the stability breakdown in region IV. Reproduced with permission.^72^ Copyright 2014, American Chemical Society.

The other fluid properties that should be considered for creating well‐defined droplets are ink viscosity, surface tension, density, and inertia. In particular, surface tension and viscosity are the primary physical properties that determine the shape and droplet‐tail of in‐flight droplets, and satellite droplet formation. For further normalized analysis, the primary four dimensionless numbers, the Reynolds number (*Re*), the Weber number (*We*), the capillary number (*Ca*), and the Ohnesorge number (*Oh*), are calculated^71^

(1) $$Re=\frac{\mathrm{inertial}\;\mathrm{force}\;}{\mathrm{viscous}\;\mathrm{force}\;}=\frac{\rho dv}{\eta }$$

(2) $$We=\frac{\mathrm{inertial}\;\mathrm{force}}{\mathrm{surface}\;\mathrm{tension}\;\mathrm{force}}=\frac{\rho v^{2}d}{\gamma }$$

(3) $$Ca=\frac{\mathrm{viscous}\;\mathrm{force}\;}{\mathrm{surface}\;\mathrm{tension}\;\mathrm{force}\;}=\frac{\eta v}{\gamma }$$

(4) $$Oh=\frac{\sqrt{We}\;}{Re}=\frac{\eta }{\sqrt{\gamma \rho d}}$$

where ρ is the ink density, *d* is the nozzle diameter, *v* is the velocity of the ink, η is the viscosity of the ink, and γ is the surface tension. These dimensionless parameters offer a window of jetting conditions for general inks regardless of composition. For example, if the jetting is dominated by a high‐viscosity ink, a large value of *Oh* (>1) is extracted, whereas unstable jetting can be observed if *Oh* has a small value (<0.1)^72^ (**Figure** **4**). In other words, the Ohnesorge number actually describes the compatibility (“ink printability”) between the ink chosen for the printing process (hence its dependence on γ, ρ, and η) and the equipment used for droplets generation (hence its dependence on *d*). It should be noted that the inverse Ohnesorge number (*Z* = *Oh* ^−1^) is also widely used (1 < *Z* < 10). *Ca* is typically determined by the diameter of the nozzle and does not depend on the characteristic length. In commercial inkjet printing systems, typical nozzle diameters are in the range of 20–60 µm, a tolerable ink viscosity range is 0.5–40 cP and a tolerable surface tension range is 20–70 dyne cm^−1^.

### 2.2 Printable Semiconductor Materials

#### 2.2.1 Organic Semiconductors

Over the past decades, high‐performance organic semiconductors have led the field of inkjet printable semiconductors due to their low‐temperature processability, excellent compatibility with flexible platforms, and good solubility in organic solvents with low‐viscosity, which are key advantages of the inkjet printing process.^12^, ^16^, ^73^, ^74^, ^75^, ^76^, ^77^, ^78^, ^79^, ^80^, ^81^, ^82^, ^83^ Organic semiconducting materials can be largely categorized as soluble 1) small molecules or 2) polymers. Depending on the relative position of the highest occupied molecular orbital and the lowest unoccupied molecular orbital orbitals with respect to the Fermi level of the metal used to fabricate the source and drain contacts, the p‐type or n‐type character of the organic semiconductor can be determined, which facilitate hole and electron injection, respectively. For practical complementary metal‐oxide‐semiconductor (CMOS) applications, both types of semiconductors need to be developed.

Because the electrical performance and environmental stability of solution‐processed organic TFTs (OTFTs) are relatively poor compared with those of vacuum‐processed TFTs, many research groups have introduced highly ordered π–π stacking to provide enough molecular orbital overlap for better charge transport between adjacent molecules. In this regard, fused ring compounds with a strong planar conjugated structure have been widely used as promising organic semiconductors. Since the 2000s, many researchers have extensively reported high‐performance OTFTs with acenes, such as rubrene, anthracene, tetracene, and pentacene, that exhibit high‐carrier‐mobility.^84^, ^85^, ^86^ Although, pentacene is one of the most widely used organic semiconductors due to its competitive carrier mobility from strong intermolecular interactions, the poor solubility of typical acenes in organic solvents is a bottleneck to their use in printed electronics. In this session, high‐performance solution‐processable organic semiconductors which can be potentially used for realizing inkjet‐printed electronics would be introduced. Then, we will discuss the effort to transpose these results for enhancing the electrical performance of fully inkjet‐printed OTFTs in detail.

**Figure 5:**
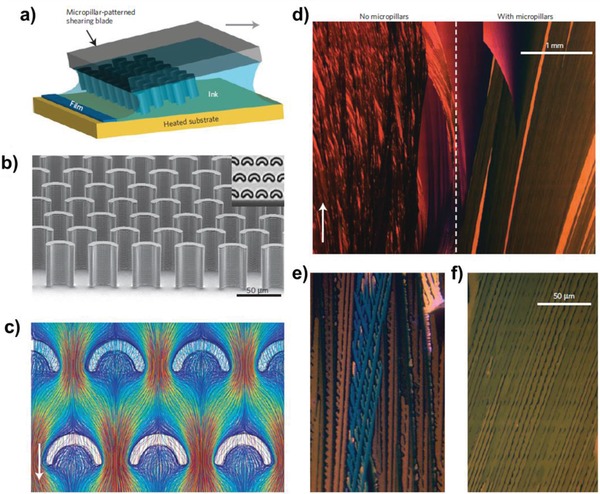
Fluid‐flow‐enhanced crystal growth. a) Schematic of solution shearing using a micropillar‐patterned blade. The arrow indicates the shearing direction. b) A scanning electron micrograph of a micropillar‐patterned blade. Inset: Top view of the micropillars under an optical microscope. The pillars are 35 µm wide and 42 µm high. c) Streamline representation of simulated fluid flow around the micropillars. The arrow indicates the flow direction. The streamlines are colour coded to indicate the scale of velocity (mm s^−1^), ranging from 0 (deep blue) to 1.3 mm s^−1^. d–f) Cross‐polarized optical micrograph of a TIPS‐pentacene film coated from its mesitylene solution with (d) (right), (f) and without micropillars (d) (left), (e) at a shearing speed of 0.6 mm s^−1^. Reproduced with permission.^98^ Copyright 2013, Springer Nature.

To address aforementioned issue, there have been many efforts to introduce functionalized groups.^87^, ^88^, ^89^ Anthony et al. reported 6,13‐bis(triisopropylsilylethynyl)pentacene (TIPS‐pentacene) substituted at the C6 and C13 positions with silicon alkynyl, resulting in much improved solubility in aromatic solvents.^87^, ^90^ As TIPS‐pentacene has also shown promising electrical performance and environmental stability, for the last two decades, studies on the optimizations of film formation, high‐k dielectric, contact engineering, and device structure have been extensively reported to realize high‐performance printed OTFTs.^67^, ^91^, ^92^, ^93^, ^94^, ^95^ Additionally, aligned crystalline domains of TIPS‐pentacene have been extensively investigated to achieve high‐mobility by spatially confining the channel area. For example, Giri et al. reported strained polymorphologys of TIPS‐pentacene using blade‐printing with a controlled shear strain applied during growth that allows the shortest π–π stacking distance enhancing the hole mobility up to 4.6 cm^2^ V^−1^ s^−1^ under optimum growth conditions with solution processing.^96^ Bao and co‐workers steadily developed a methodology to improve carrier transport by confinement in the lateral direction with consideration of ink wettability.^96^, ^97^ As a follow‐up work, Diao et al. presented the large‐area fluid‐flow‐enhanced crystal growth of TIPS‐pentacene using a micropillar‐patterned printing blade, which allows highly controlled morphologies of TIPS‐pentacene (**Figure** **5**).^98^ During the solution‐shearing process with the designed blade, crystal nucleation was engineered to deliver nonequilibrium single‐crystalline domains with of millimeter‐wide and centimeter‐long dimensions, resulting in a mobility up to 11 cm^2^ V^−1^ s^−1^. This strategy was also utilized to improve bulk polymer crystallization without the issue of microphase separation to realize high‐performance all‐polymer solar cells.^99^

**Figure 6:**
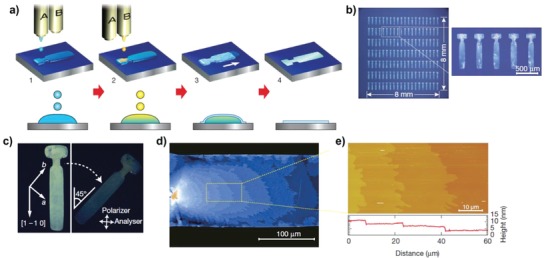
Inkjet printing of organic single‐crystal thin films. a) Schematic of the process. Antisolvent ink (A) is first inkjet‐printed (step 1), and then solution ink (B) is overprinted sequentially to form intermixed droplets confined to a predefined area (step 2). Semiconducting thin films grow at liquid–air interfaces of the droplet (step 3), before the solvent fully evaporates (step 4). b) Micrographs of a 2037 array of inkjet‐printed C8‐BTBT single‐crystal thin films. c) Crossed Nicols polarized micrographs of the film. d) Expanded micrograph of the film, showing stripes caused by molecular‐layer steps. e) Atomic force microscopy image and the height profile (below) showing the step‐and‐terrace structure on the film surfaces. Reproduced with permission.^100^ Copyright 2011, Springer Nature.

Tremendous progress in inkjet‐printed organic semiconductors was achieved in 2011 by Minemawari et al.^100^ 2,7‐Dioctyl[1]benzothieno[3,2‐b][1]benzothiophene (C8‐BTBT) can be highly crystallized at the liquid–air interface of an antisolvent with the main solvent using a dual‐shot printing method. This strategy enabled confined single‐crystal thin‐film growth patterned by spatially engineered surface energy, as shown in **Figure** **6**. From the results, high‐average and maximum mobilities of 16.4 and 31.3 cm^2^ V^−1^ s^−1^ were achieved, respectively. This dual‐shot printing with an antisolvent is feasible only in inkjet printing. As promising high‐performance small molecule organic semiconductors, triethylsilylethynyl anthradithiophene (TES‐ADT) or difluorinated (di‐F) TES‐ADT semiconductors blended with polymers formed by spin‐casting, and blade‐printing for contact‐induced nucleation also exhibited comparable electrical performance to their TIPS‐pentacene counterparts.^101^, ^102^, ^103^, ^104^

Polymer semiconductors are also regarded as promising candidates for use in printed OTFTs due to their good solubility in inkjet printable solvents. On the basis of their good flexibility and low‐temperature processability on large‐area substrates, further improvements in the mobility and switching performance have been widely investigated for application in practical flexible TFT applications. To meet this requirement, material design strategy is focused on 1) reduction of the energy barrier between adjacent molecules by allowing enough overlap between the molecular orbitals and 2) the orientation of polymer molecules considering the direction of π–π stacking. For the last two decades, regioregular poly(3‐hexaylthiophene) (RR‐P3HT) has been one of the most attractive materials. In particular, by optimizing the average molecular weight (*M*_w_), the length of the alkyl chain, and the solvent for dissolving RR‐P3HT, solution‐processed polymer TFTs were produced with a mobility close to 0.1 cm^2^ V^−1^ s^−1^,^105^ and the mobility has gradually increased up to 0.4 cm^2^ V^−1^ s^−1^.^106^, ^107^ Importantly, polythiophene‐based random copolymers exhibited a greatly enhanced mobility of 1.37 cm^2^ V^−1^ s^−1^ by introducing localized aggregates for better connectivity between polymer chains.^108^ Naphthalene‐based n‐type semiconducting polymers are also promising materials for implementing polymer CMOS circuits. The semiconductor developed by Yan et al. exhibited a mobility up to 0.85 cm^2^ V^−1^ s^−1^ for printed TFTs,^109^ and Izuhara and Swager reported drastically improved mobility as high as 3.4 cm^2^ V^−1^ s^−1^.^110^ Further investigation on efficient surface treatments onto the gate dielectric layer to introduce π–π stacking parallel to the desired channel as well as the development of environmentally stable polymer materials is highly needed to address the inherently low‐carrier‐mobility and on/off ratio.^110^

Recently, donor–acceptor (D–A)‐type polymer semiconductors allowing enhanced intermolecular carrier hopping have exhibited superior mobilities over 10 cm^2^ V^−1^ s^−1^.^111^, ^112^, ^113^ Kim et al. reported a synthesized polymer based on thienoisoindigo‐naphthalene with a hole‐mobility of 14.4 cm^2^ V^−1^ s^−1^,^111^ and a hole mobility of 17.8 cm^2^ V^−1^ s^−1^ at *V*_GS_ = *V*_DS_ = −150 V was achieved with the diketopyrrolopyrrole based semiconductor by optimizing the side‐chain geometry to determine the best conditions for charge transport.^112^ Nketia‐Yawson et al. suggested a novel strategy to synthesize a highly planar poly(4‐(4,4‐bis(2‐ethylhexyl)‐4H‐silolo[3,2‐b:4,5‐b′]dithiophen‐2‐yl)‐7‐(4,4‐bis(2‐ethylhexyl)‐6‐(thiophen‐2‐yl)‐4H‐silolo[3,2‐b:4,5‐b′]dithiophen‐2‐yl)‐5,6‐difluorobenzo[c][1,2,5]thiadiazole) (PDFDT) based on the dithienosilole and difluorobenzothiadiazole moieties by incorporating a high‐k dielectric of poly‐(vinylidenefluoride–trifluoroethylene) to improve coplanarity of the polymer backbone and induce strong π–π interactions.^114^ This hole mobility is far superior to that of amorphous silicon field‐effect transistors (FETs) and comparable to vacuum‐deposited small molecule/single‐crystal organic FETs. In regard to n‐type semiconductors, the strategy introducing alkyl or fluorinated alkyl moieties on the N atoms of naphtalenediimides is widely used because of its relatively better solubility and air stability.^115^ Also, in the latest result, excellent thermal durability was reported in the solution‐processed OTFTs based on a dimerized naphtho[2,3‐b]thiophenediimide derivatives with branched alkyl chains.^116^

As a different strategy, Hamilton et al. reported the blending of high‐mobility TIPS‐pentacene and TES‐ADT with inert and semiconducting polymers having good film formation ability.^102^, ^117^, ^118^, ^119^ By employing amorphous p‐type poly(triarylamine) as a blending polymer, the carrier mobility of the blended semiconducting films was improved achieving significantly better film uniformity, whereas much lower mobility was exhibited in the blended counterparts with an insulating polymer matrix, poly(a‐methyl styrene).^117^ In 2012, Smith and co‐workers proposed a similar approach to improve the electrical performance in a blended system of acene and polymer without affecting the phase separation. This study emphasizes that imparting efficient conduction between acene‐rich regions is the most promising strategy to improve the effective carrier mobility and reproducibility. The TES‐ADT FETs blended with poly(dialkyl‐fluorene‐co‐dimethyl‐triarylamine) (PF‐TAA) exhibited a hole mobility over 5 cm^2^ V^−1^ s^−1^ and a large on/off ratio of 10^6^ due to the improved crystallinity of the TES‐ADT thin films.^102^ In addition, introduction of the molecular p‐type dopant into the blends drastically improved the hole‐transport properties.^118^ These strategies can also be utilized to realize high‐performance inkjet‐printed OTFTs. Recently, a blade coating method was also utilized in a blended system of conjugated small molecules and insulating polymer materials to produce single‐crystal OTFTs.^120^ The electrical characteristics were significantly improved by optimizing the crystallinity without imparting geometrical defects, indicating the importance of the printing conditions.

#### 2.2.2 Metal Oxide Semiconductors

Metal oxide materials have been highlighted as promising semiconductors exhibiting a superior carrier mobility, on/off ratio, and low‐voltage operation. Because traditional methods to produce high‐performance oxide films using vacuum processes such as atomic layer deposition or sputtering are not suitable for printed electronics or large‐area applications, ink‐type metal oxide materials have been widely investigated by optimizing the elemental composition.^121^, ^122^, ^123^, ^124^ Postannealing at high‐temperatures over 400 °C results in the complete conversion of chemical precursors into metal–oxide–metal (M—O—M) bonds, which limits the choice of substrates to rigid silicon or glass substrates.^122^, ^125^, ^126^, ^127^ For example, indium gallium zinc oxide (IGZO) is one of the most popular channel materials due to its amorphous nature and high operational stability, but low‐temperature‐processed printed IGZO TFTs have not been widely reported because high‐temperature processing (at least 300–400 °C) is required to completely convert tin and zinc‐based channel compositions for competitive electrical performance.^128^, ^129^, ^130^ In this regard, in the field of printed electronics, tremendous efforts for preparing solution processed metal oxide systems at low‐temperature (≈250 °C) to be implemented on flexible substrates have been reported to benefit printing technology.^131^, ^132^, ^133^, ^134^ To satisfy thermal budgets of flexible platforms including a substrate and underlying deposited layers, the optimized design of metal oxides requires consideration of the trade‐offs between the electrical performance and thermal tolerance. In the recent reports, representative strategies to achieve low‐temperature processing are the introduction of 1) aqueous solutions, i.e., metal nitrate and ammine‐hydroxo, which do not require the use of toxic volatile solvents;^134^, ^135^, ^136^, ^137^ 2) combustion chemistry;^131^, ^138^, ^139^, ^140^ and 3) alternative annealing for photoactivation, achieving high‐performance (*µ*_eff_ > 10 cm^2^ V^−1^ s^−1^) at lower annealing temperatures, i.e., below 250 °C.^141^, ^142^ Additionally, many efforts to avoid the use of high‐cost and rare materials, such as indium, have been reported to realize long‐term sustainability. In 2013, novel tin dioxide (SnO_2_) gel‐like precursors for high‐performance semiconducting layer formation were reported as SnO_2_ has an outstanding intrinsic mobility, a wide‐bandgap, and a relatively low melting temperature compared to Zn and In.^143^, ^144^ Moreover, a threefold boost in the saturation mobility was achieved with the gel‐like phase formed by adding NH_4_OH. Although fully inkjet‐printed SnO_2_ TFTs were realized with a sol–gel deposited zirconium dioxide (ZrO_2_) gate dielectric exhibiting a competitive mobility of 11 cm^2^ V^−1^ s^−1^, unfortunately high‐temperature processing over 400 °C was still required, which cannot be utilized in flexible printed systems. Recent results for the realization of flexible printed metal oxide TFTs are introduced in Section 3.

In contrast to well‐established n‐type oxide semiconductors, their p‐type counterparts still find difficulty in achieving high electrical performance and reliability for the implementation of practical complementary electrical applications, such as solar cells.^145^ There are two widely accepted strategies: employing 1) doped n‐type semiconductors,^146^, ^147^ and 2) oxide semiconductors that can deliver p‐type conduction.^148^, ^149^, ^150^ Because of the relatively poor stability and reproducibility of the self‐compensation of the former approach, high‐performance solution‐processable p‐type metal oxide semiconductors synthesized with sol–gel precursors such as Cu_2_O, CuO, and ZnO have been intensively developing to improve the intrinsic hole‐mobility.

#### 2.2.3 Single‐Walled Carbon Nanotubes and 2D Semiconductors

**Figure 7:**
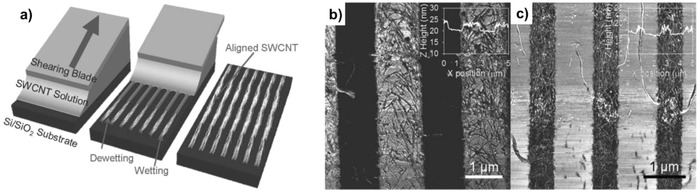
SWCNT solution shearing technique. a) Schematic depiction of SWCNT alignment using solution shearing. AFM phase image of sheared SWCNTs on b) 1.2 µm and c) 0.6 µm wide solvent‐wetting regions. The images show better alignment of SWCNTs on the 0.6 µm wide solvent‐wetting strips. The insets are height profiles obtained using topography images. Reproduced with permission.^156^ Copyright 2015, Wiley‐VCH.

Single‐walled semiconducting CNTs (SWCNTs) have attractive advantages for realizing high‐performance TFTs on flexible substrates due to their remarkable mechanical robustness, high‐carrier‐mobility, low‐temperature, and large‐area processability. In recent results, beyond the electrical performance, many efforts to convert the polarity of SWCNTs from p‐type to n‐type using effective doping strategies have been reported for CMOS applications.^151^, ^152^, ^153^, ^154^, ^155^ The high electrical performance of SWCNTs promises their utilization in integrated logic and display backplane driving circuits. In printed CNT TFTs, SWCNT networks have been widely used for channel formation rather than single formation, and there has been great progress over the last few years 1) to separate the different types of metallic and semiconducting CNTs and 2) to form well‐aligned CNT channels between source/drain (*S*/*D*) electrodes, for example, by the shearing process (**Figure** **7**).^152^, ^156^, ^157^, ^158^, ^159^, ^160^ Additionally, CNT networks have been widely utilized in stretchable electronics, such as stretchable electrodes, TFTs, and logic gates.^161^, ^162^

**Figure 8:**
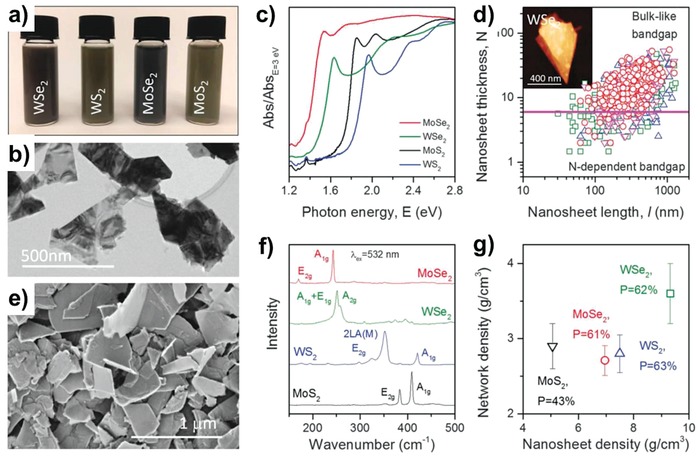
Basic characterization of nanosheets and nanosheet networks. a) Photo of dispersions of MoS_2_, MoSe_2_,WS_2_, and WSe_2_ (*C* ≈ 0.2 mg mL^−1^). b) Typical TEM image of liquid‐exfoliated WSe2 nanosheets. c) Optical absorption spectra (extinction minus scattering) measured on nanosheet dispersions (*C* ≈ 0.005 mg mL^−1^). d) Plot of nanosheet length versus thickness (layer number *N*) for all materials. The horizontal line approximately separates thinner nanosheets with N‐dependent bandgap from thicker ones with bulk‐like bandgap. Inset: Typical AFM image. e) Typical scanning electron microscopy (SEM) images of a sprayed network of WSe_2_ nanosheets. f) Raman spectra measured on networks of all four materials. g) Measured network density plotted versus nanosheet density; the resultant porosity values *P* are indicated. Reproduced with permission.^179^ Copyright 2017, American Association for the Advancement of Science.

Mechanically flexible and optically transparent 2D materials have also emerged due to their good intrinsic mobility in atomically thin channel layers.^163^, ^164^, ^165^, ^166^, ^167^ Specifically, transition metal dichalcogenides (TMDCs) including molybdenum disulfide (MoS_2_), tungsten disulfide (WS_2_), and tungsten diselenide (WSe_2_) have led the field of 2D semiconductors due to their interesting properties such as tunable bandgap energy controlled by the number of layers, and spin‐valley physics from the strong internal magnetic field.^167^, ^168^, ^169^, ^170^, ^171^ Note that although there are many approaches to widen the bandgap energy in graphene, for example, by introducing patterning in nanoribbon structures or chemical doping due to its semimetallic properties and zero bandgap energy,^172^, ^173^, ^174^, ^175^ the consequential results required more enhanced electrical performance without degradation of the mobility. For large‐area and cost‐effective fabrication, the field of 2D electronics has also extensively utilized solution‐processes or printing technologies. At the first onset in 2012, inkjet‐printed graphene electronics were reported using liquid‐phase exfoliated graphene dispersed in N‐methyl‐2‐pyrrolidone (NMP) on a SiO_2_/Si substrate.^176^ Since, various types of graphene inks have been widely utilized for transparent FET applications or transparent electrodes fabricated by rod‐coating or inkjet printing. However, additional postprocesses, such as high‐temperature or intensive pulse‐light annealing, are necessary to remove stabilizing polymers. Since 2013, reduced graphene oxide (rGO) dispersed in organic solvents, such as ethylene glycol with sodium dodecylbenzenesulfonate, has also been widely used in semiconducting channels and electrodes to realize transparent and flexible all‐carbon TFTs due to the relatively low annealing temperature and facile postprocessing. The fabricated rGO TFTs exhibited much improved switching characteristics including an on/off ratio of 10^4^ and mobility of 8 cm^2^ V^−1^ s^−1^.^177^, ^178^ Recently, TMDC inks which are mostly MoS_2_ and WSe_2_ dispersed in NMP have also been reported for 2D TFT applications. Specifically, Kelly et al. reported various 2D semiconductor inks of MoS_2_, MoSe_2_, WS_2_, and WSe_2_ dispersed in NMP for channel layer formation in fully inkjet‐printed 2D TFTs (**Figure** **8**).^179^ Although their electrical characteristics were still not impressive compared to those of TMDCs fabricated by a conventional mechanical exfoliation, it opened a new opportunity to realize fully printed 2D electronics that are similar to the all‐inkjet‐printed OTFTs published for the first time in 2000 by Sirringhaus et al.^66^ In addition, to realize insulating properties, hexagonal boron nitride (*h*‐BN) nanosheet inks dispersed in isopropyl alcohol (IPA) or dimethylformamide (DMF) with polycarbonate were deposited using a spray‐coating or screen‐printing process to form heterostructures,^180^, ^181^ but critical technical issues, such as film uniformity, dielectric constant loss, and insulating performance, remain to be implemented in fully printed TFT applications.

## 3 Recent Process in Inkjet‐Printed Thin‐Film Transistors

This section describes recently achieved progress in inkjet‐printed TFTs in terms of their electrical performance. In particular, their flexible applications using low‐temperature processing are highlighted which can be utilized in next‐generation low‐cost wearable electronics. Based on the active materials mentioned above, this section is organized into four main parts. Regarding inkjet‐printed organic TFTs, representative recent progress to improve the 1) electrical performance, 2) degree of integration, and 3) emerging applications is introduced. Subsequently, efforts to address the general requirements of inkjet printing to be fully utilized for oxide TFTs are discussed. As low‐dimension materials have attracted much attention, their research has also been extended to the field of printed electronics. In this manner, recent achievements of fully or partially inkjet‐printed TFTs with 2D materials are introduced. In addition, inkjet‐printed mechanical switches, i.e., MEM relays, regarded as low‐power consumption switches, are introduced in the section concerning emerging switching devices.

### 3.1 Organic Thin‐Film Transistors

Over the last few decades, a variety of inkjet‐printed organic TFTs and their logic applications have been reported because of the aforementioned significant developments in organic semiconductor inks. From early 2008, inkjet‐printed components were partially used to fabricate thin‐film devices due to the difficulty in depositing optimized inkjet‐printed layers sequentially, for example, only semiconducting layers or electrodes were formed by inkjet printing.^182^, ^183^, ^184^, ^185^, ^186^, ^187^ As the inkjet printing technique has matured, fully printed single devices or logics such as one‐diode connected inverters or ring‐oscillators have been widely reported.^67^, ^95^, ^188^, ^189^, ^190^ Novel approaches to improve their electrical performance including high‐mobility, low‐operation voltage, and sharp switching with high‐yield have been extensively studied. Recently, many research groups have tried to utilize printed OTFTs for highly integrated printed analog, mixed‐signal, and digital circuit applications, which require guaranteed excellent device‐to‐device uniformity and high‐resolution printing to enable high‐speed operation.^191^, ^192^, ^193^, ^194^, ^195^, ^196^

**Figure 9:**
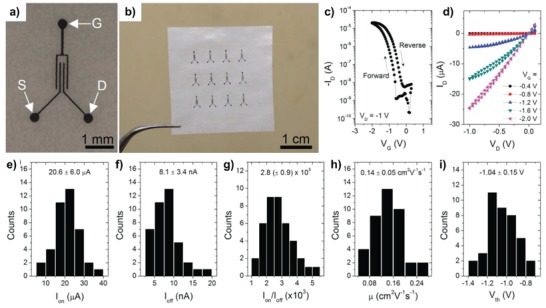
a) OM image of screen‐printed graphene for gate (G), source (S), and drain (D) electrodes on glassine paper. b) Photograph of a 3 × 4 array of organic TFTs. c,d) Representative transfer and output characteristics, respectively; the transfer curve was collected with a voltage sweep rate of 50 mV s^−1^. e–i) Histograms of device metrics for 40 devices (4 batches × 10 devices), including the on‐current (*I*_on_), off‐current (*I*_off_), on/off‐current ratio (*I*_on_/*I*_off_), charge carrier mobility (*µ*), and threshold voltage (*V*_th_), respectively. Reproduced with permission.^198^ Copyright 2015, Wiley‐VCH.

**Figure 10:**
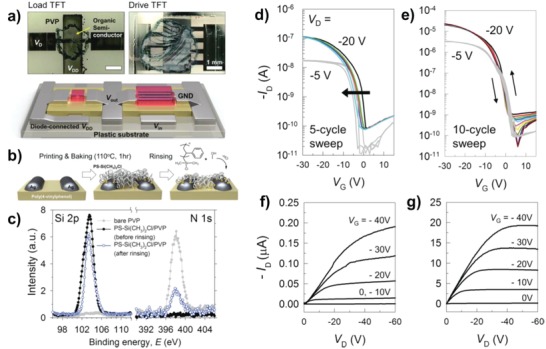
a) Optical and schematic images of an all‐inkjet‐printed inverter using two p‐type OTFTs. b) Schematic diagrams of the PS brush treatment procedure on the PVP gate dielectric and Ag *S*/*D* electrodes (20 nm thick PS‐Si(CH_3_)_2_Cl layers were inkjet‐printed to cover all the UV/O_3_‐treated Ag electrodes/PVP gate dielectrics. Subsequently, they were annealed at 110 °C for 1 h and then rinsed with an excess of toluene). c) XPS profiles for Si 2p and N 1s intensity of the bare and PS brush treated PVP gate dielectrics before and after rinsing with toluene. All‐inkjet‐printed p‐type OTFT d,e) transfer (*V*_DS_ of −5 and −20 V under *V*_GS_ multisweeps from 20 to −30 V) and f,g) output characteristics d,f) without and e,g) with a PS brush interlayer. Reproduced with permission.^95^ Copyright 2013, Wiley‐VCH.

For better electrical performance, one promising strategy is the use of solid electrolytes as the gate dielectric. Electrical double layers, regarded as nanometer‐thick capacitors, can be formed at the interfaces of gate‐dielectric and dielectric‐semiconductors, which allow low‐voltage operation with a unit capacitance over 1 µF cm^−1^ (**Figure** **9**).^197^, ^198^ The other approach is the introduction of functionalized layers at the interfaces related to organic semiconductor formation. Because of the temperature budget for sintering *S*/*D* electrodes and the chemical dissolution of organic semiconductors by sequentially dropped inks for *S*/*D* electrode formation, bottom‐gate and bottom‐contact structures are typically employed for fully printed OTFT fabrication that causes much higher contact barriers.^95^, ^199^, ^200^ For the better electrical performance of fully inkjet‐printed OTFTs regarding carrier mobility, environmental stability, SS, and on/off ratio for a wide range of applications, two critical issues must to be addressed: one is the uniform deposition of organic semiconductors, and the other is lower carrier injection barriers between the *S*/*D* electrodes and organic channel layer. To meet these requirements, surface‐energy matching of semiconductor inks with underlying dielectric and *S*/*D* electrodes should be guaranteed to deposit the inkjet‐printed organic channel layer over the entire underlying layer. Generally, self‐assembly monolayer (SAM) treatments are used to provide sufficient surface properties using solution immersion or vapor exposure,^201^, ^202^ which have been conducted only on gate dielectric or bottom electrodes, but this approach does not have the advantages of inkjet printing in terms of the fast, selective, and large‐area processability. In this regard, Chung et al. suggested a single‐step surface treatment that is effective for both inkjet‐printed cross‐linked polymer dielectric and Ag *S*/*D* electrodes simultaneously (**Figure** **10**).^95^ By depositing an end‐functionalized polystyrene (PS) layer onto the underlying layers, well‐ordered crystals of organic semiconductors and reduced carrier‐injection barriers were allowed, resulting in improved carrier transport abilities and contract properties. The PS could be chemisorbed onto ultraviolet ozone (UVO)‐treated underlying layers which provided excellent solvent resistance from the sequentially dropped organic semiconductor inks.^203^ Recently, this methodology has also been utilized on conductive polymer (PEDOT:PSS) electrodes to realize high‐performance fully transparent OTFTs and inverters.^200^ The introduction of a chemisorbed functionalized organic layer where channel layers are formed can be one of the most efficient approaches to improve the electrical characteristics, especially for highly crystallized organic semiconductors and lower contact resistance.^199^

**Figure 11:**
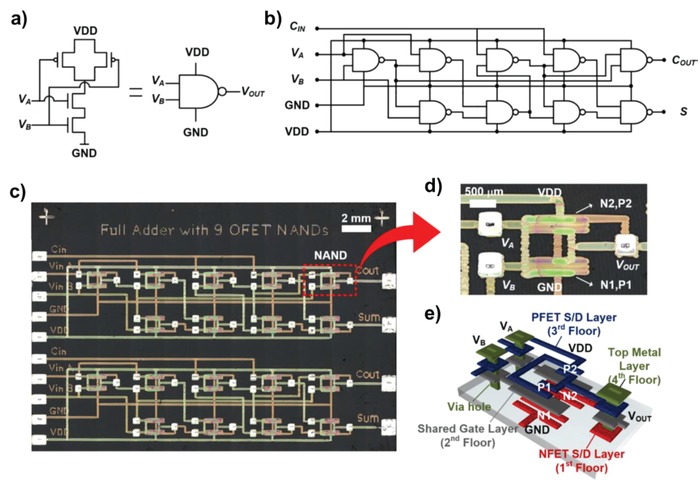
a) 3D schematic cross‐section of the 3D‐complementary OFETs (3D‐COFET) with a bottom‐gate p‐type FET (PFET) vertically stacked on a top‐gate n‐type FET (NFET). b) Top view of 56 pairs of 3D‐COFET inverters fabricated by inkjet printing on a substrate. c) Microscope images of a 3D‐COFET inverter and d) printed active regions (white dotted areas) observed from the bottom (NFET) and the top (PFET) FETs by optical microscopy (scale bars = 200 µm). e) Transfer characteristics (|*I*_DS_| vs *V*_GS_) and f) output characteristics (|*I*_DS_| vs *V*_DS_ with 2 V step *V*_GS_) of the NFET (red, left graphs) and the PFET (blue, right graphs). Reproduced with permission.^207^ Copyright 2016, American Chemical Society.

**Figure 12:**
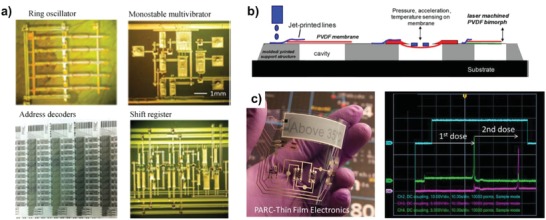
a) Micrographs of various printed digital circuits. b) Illustration of the piezoelecric pressure sensor and accelerometer fabrication. c) Printed temperature label with memory and Writing temperature dose signals into memory. Reproduced with permission.^42^ Copyright 2015, IEEE.

The other impressive progress is the improvement of the degree of integration with high device yield. Because this requirement is essential to realize highly reliable integrated circuits, Sowade et al. analyzed the most critical parameters affecting the device yield during the inkjet printing process.^204^ By optimizing mainly the print resolution, they reported that the OTFT arrays showed a high process yield up to 82%.^205^ Kwon et al. reported 3D integrated circuits based on OTFTs connected by via‐hole sharing gate electrodes, and the NAND gate was implemented with 100% yield.^206^, ^207^ By vertically stacking p‐type and n‐type OTFTs with the bottom‐gate and top‐gate respectively, a high transistor density of 4.4 TR mm^−2^ was achieved, which could provide an attractive solution to address the inherent challenge of low‐integration density due to the relatively poor resolution, therefore they proposed a key pathway for the realization of organic digital integration circuits (**Figure** **11**). In contrast to the vertical stacking strategy, Mahajan et al. also provided a solution for improving printing‐resolution by exploiting the capillary force laterally.^208^ Consequently, by using the methodology of self‐aligned capillarity‐assisted lithography, the submicrometer patterning and self‐alignment of channel layers in side‐gating OTFTs was enabled, resulting in a short gate distance of 4.6 µm and channel length of 1.5 µm.^209^ The use of electrolyte‐gate dielectric and high‐resolution patterning facilitated improved electrical characteristics of the P3HT OTFTs.^198^ In addition, there have been many efforts to minimize the channel length using surface‐energy engineering, which have also helped to understand high‐resolution TFTs.^210^, ^211^ With these efforts, as printing technologies have been matured, large‐area applications on flexible substrates including displays,^212^ logics,^213^ and biosensors^214^, ^215^ have been reported with high yields. Beyond the previous achievements, printed organic electronics can be important players for leading IoT generation because of their additive functionality and low‐cost manufacturing with abundant materials, which enables promising mass‐customization. Additionally, the excellent flexibility, low‐temperature processing, and lightweight nature of organic electronics can provide a promising route for realizing smart sensor systems, such as blast dosimeters and smart labels including temperature and humidity sensors, as well as integrated flexible circuits based on printed TFTs (**Figure** **12**).^42^, ^216^, ^217^

### 3.2 Metal Oxide Thin‐Film Transistors

**Figure 13:**
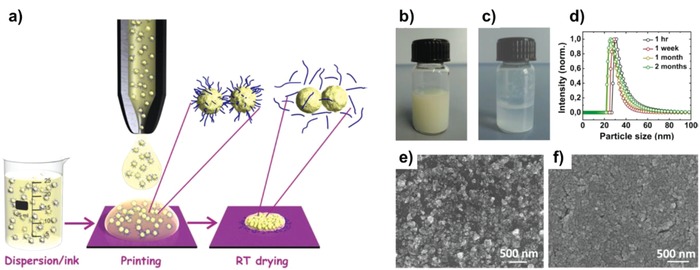
a) Schematic representation of the chemically controlled destabilization and flocculation process of the printed nanoink droplets. The NaCl loaded semiconducting oxide nanoinks show spontaneous stabilizer removal from the nanoparticle surface during the ink drying process. Stability of the In_2_O_3_ nanoinks with PAANa as the stabilizing ligands and different halide ion concentration. b) In_2_O_3_ nanoink with 20 × 10^−3^ m NaCl, after 2 months of ink preparation. c) In_2_O_3_ nanoink with 50 × 10^−3^ m NaCl, after 1 h of ink preparation. d) DLS particle size distribution of the In_2_O_3_ nanoink with 20 × 10^−3^ m NaCl concentration, as a function of the elapsed time. Surface morphology of the printed In_2_O_3_ thin films, SEM images showing surface topography of the printed droplets from nanoparticulate inks that contain e) no NaCl and f) 20 × 10^−3^ m NaCl, respectively. Reproduced with permission.^132^ Copyright 2015, American Chemical Society.

**Figure 14:**
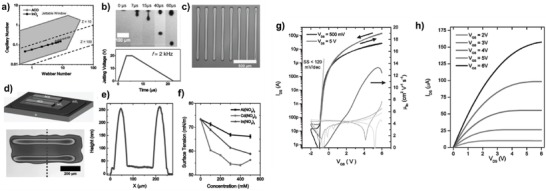
a) ACO inks plotted on a Capillary (Ca) number/Weber (We) number diagram with empirically determined jettable window shaded in green and *Z* numbers 10 and 100 indicated by dashed lines. b) Stroboscopic snapshots of ACO droplets jetted from the piezoelectric print head by the corresponding jetting waveform. c) Array of inkjet‐printed transparent conductive lines. d) Diagram of oxide TFT structure with printed semiconductor and printed *S*/*D* electrodes with matching optical micrograph. e) Height profile of InOx‐printed transistor measured transverse to ACO electrodes. f) Surface tension of aqueous inks formulated from aluminum, indium, and cadmium nitrate salts. g) Transfer characteristics with linear mobility shown and h) output characteristics of aqueous InOx‐printed transistor with printed ACO contacts processed at ≤250 °C. Reproduced with permission.^133^ Copyright 2017, Wiley‐VCH.

**Figure 15:**
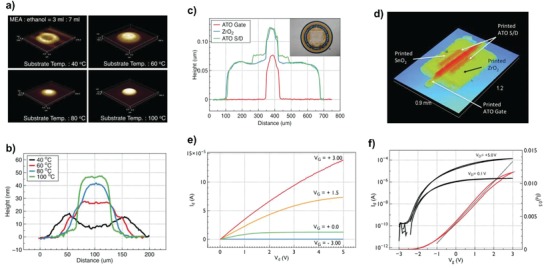
The morphology of isolated ZrO_2_ droplet patterns as a function of substrate temperature on PMMA‐coated substrates: a) surface profiles and b) typical cross‐sectional profiles. c) Cross‐sectional profile of the fabricated TFTs. The inset shows the fabricated TFTs. d) Surface profiles of the TFT fabricated on a glass substrate. The inset shows the optical image. Representative TFT characteristics: e) *I*_d_–*V*_d_ and f) *I*_d_–*V*_g_ (left axis) and *I*_d_^0.5^–*V*_g_ (right axis). Reproduced with permission.^221^ Copyright 2015, Wiley‐VCH.

As the demands of transparent displays increase, transparent oxide TFTs are regarded as the most attractive driving component to fulfill the required electrical performance and stability, thus offering better promise than their organic counterparts.^141^, ^159^, ^218^, ^219^, ^220^ In particular, solution‐processable oxide materials can facilitate the implementation of large‐area transparent electronics. So, inkjet printing has been also extensively employed to fabricate transparent metal‐oxide TFTs, specifically for high‐performance channel layers formation. However, there are three major issues to fully bring the advantages of inkjet printing for practical CMOS applications: 1) high processing temperature, 2) difficulty in the realization of fully inkjet‐printed oxide TFTs, and 3) relatively inferior electrical performance of p‐type oxide TFTs. After low‐temperature solution‐processed metal‐oxide TFTs were reported via combustion processing which allows plastic‐compatible temperature annealing at temperature as low as 200 °C for a variety of metal oxide materials,^131^ the study of inkjet‐printed metal oxide TFTs has been intensively conducted to implement a low‐thermal budget. Low‐temperature combustion processing has also been utilized for low‐voltage‐operation IGZO TFTs with hafnia self‐assembled nanodielectrics.^139^ In 2015, completely room‐temperature processed printed oxide TFTs and complementary inverters were reported (**Figure** **13**).^132^ Dense oxide film formation was facilitated at room temperature by optimizing the chemically controlled curing process of the nanoparticle inks. In particular, the concentration of flocculation agent (NaCl in this work) was carefully determined to realize improved long‐term stability of the inks and better nanoparticle dispersion ability. Aqueous chemistries involving sol–gel metal oxide inks are also well‐known approaches for low‐temperature processing (**Figure** **14**),^133^ which allow oxide films to be converted at 250 °C for both conductive electrodes and semiconductors. Additionally, Al doping could improve the transparency and conductivity, such that electrical and optical performances competitive to those of oxide TFTs fabricated using organic solvent‐based oxide inks were achieved. Although these strategies have not been fully utilized in inkjet printing to realize TFTs, their efforts show a promising route toward flexible metal oxide electronics. Fully inkjet‐printed oxide TFTs have not been vigorously reported due to the difficulties associated with the uniformly deposited metal oxide films using inkjet printing. To address the issue of ink wettability, which is one of the most critical factors to make well‐defined printed features, Jang et al. used ultrathin poly(methyl methacrylate) (PMMA) assistant layers with the optimized substrate temperature conditions (**Figure** **15**).^221^ This wettability switching layer provided sufficient hydrophobic surfaces, so that well‐defined patterns can be made. After printing was completed, the PMMA layers were burned‐off without affecting the electrical performance of the oxide TFTs. The solvent system was also carefully optimized to suppress the coffee‐ring effect by adding high‐viscosity surfactants, such as ethylene glycol or ethanolamine. As a similar strategy, surface‐energy patterning was reported by Li et al.^222^ By using a solvent etching method with pure solvents and oxygen plasma treatment, appropriate surface energy patterns were introduced where inkjet‐printed oxide materials could be deposited. From these results, interface engineering to deliver well‐matched surface energies with functional inks was significantly important to realizing all‐inkjet‐printed oxide electronics in this stage. The other issue to implement complementary electronics is the relatively poor performance of p‐type metal oxide TFTs. As mentioned before, there have been intensive efforts to improve the electrical performance and stability of p‐type TFTs; however, to date, satisfactory results have not been obtained. Sol–gel processed copper and nickel oxide have been widely investigated as a competitive p‐type semiconductors and utilized in inkjet‐printed oxide TFTs. Still, however, the electrical performance was relatively poor exhibiting a hole mobility below 1 cm^2^ V^−1^ s^−1^,^149^, ^150^, ^223^ so the p‐type CNT materials have been used as a hybrid system for transparent complementary applications.^224^, ^225^, ^226^

### 3.3 Single‐Walled Carbon Nanotubes and 2D Thin‐Film Transistors

For the last decade, printed or solution‐processed SWCNTs have made tremendous progress highlighting their ideal semiconducting properties. Chen et al. showed the possibility of utilizing CNTs in electronic circuits,^227^ and Franklin et al. reported sub‐10 nm gate length transistors operating at extremely low‐voltages.^228^ For further achievements, the use of well‐assembled high‐purity SWCNTs has led to much improved electrical performance. Because as‐synthesized SWCNTs also include metallic nanotubes, which result in a relatively poor on/off ratio and switching characteristics, many efforts to eliminate metallic species have been reported, such as surfactant extraction, selective chemical functionalization, and selective oxidation.^229^, ^230^, ^231^ Specifically, sorting high‐purity and large‐diameter semiconducting SWCNTs for ink preparation is one of the most important procedures for realizing high‐performance printed SWCNT TFTs economically on a large scale.^125^, ^224^, ^232^ Furthermore, due to the well‐known trade‐off between high on‐conductance and high on/off current ratios depending on residual metallic nanotubes and SWCNT densities over the percolation limit, the semiconducting layer has been numerically modeled as a finite‐sized tube percolation network.^233^, ^234^, ^235^ As described in the previous section, surface‐energy matching of semiconducting SWCNT inks with underlying printed dielectric and *S*/*D* layers is also highly necessary to enhance the contact or adhesion properties resulting in better channel formation and carrier injection. Because DMF and water are widely used as solvents to disperse SWCNTs, an increase in the surface energy of the previous formed layers by UVO or oxygen plasma treatment can improve the binding force with SWCNT inks.^236^, ^237^ In particular, printed CNT electrodes can be easily peeled off during rinsing procedures if adhesion with the underlying substrates or layers is poor.^238^

**Figure 16:**
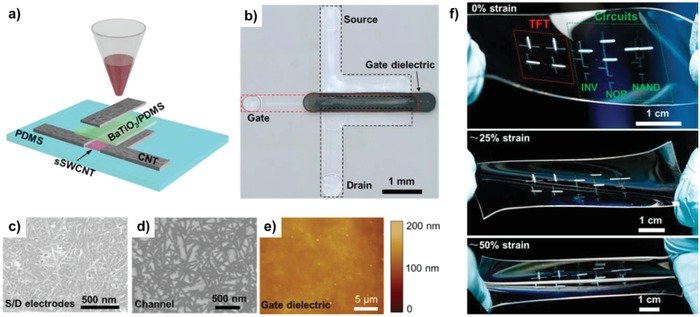
Fully printed and intrinsically stretchable carbon nanotube thin‐film transistors (TFTs) and integrated logic circuits. a) Schematic illustrating the structure of a printed stretchable TFT. Unsorted carbon nanotubes, high‐purity semiconducting single‐walled carbon nanotubes (SWCNT), and BaTiO_3_/PDMS composite are used as the source/drain/gate electrodes, channel semiconductor, and gate dielectric, respectively. b) Optical micrograph of a TFT printed on a PDMS substrate. c–e) Scanning electron micrograph of c) the carbon nanotube network in the source/drain electrodes and d) channel and e) atomic force micrograph of the BaTiO_3_/PDMS gate dielectric. f) Optical photograph of a representative sample consisting of four TFTs, a resistive load inverter, and a resistive load two‐input NOR gate and NAND gate, at tensile strains of 0% (top), ≈25% (middle), and ≈50% (bottom). Reproduced with permission.^162^ Copyright 2016, American Chemical Society.

Despite intrinsic material issues, SWCNT TFTs with good bias and light stress stability, high on/off ratios, and high on‐state current can be utilized in display applications^48^, ^239^ and small active‐matrix displays.^240^ In 2013, Javey and co‐workers reported fully printed SWCNT TFT arrays on a large‐area flexible substrate.^241^ Although gravure printing was used for the patterning process, this result showed the possibility of low‐cost and large‐area CNT TFT formation using printing techniques. The printed transistors showed mobility of a 4.3 ± 1.6 cm^2^ V^−1^ s^−1^ and an on/off ratio up to 10^5^. Moreover, to achieve a much lower operating voltage, the gate dielectric was changed from conventional polymer dielectrics or brittle oxide layers to electrolytes such as an ionic liquid. Nanometer‐thick electric double layers in the electrolyte dielectric, which is widely used in printed OTFTs, lead to high effective capacitances up to tens of µF cm^−2^. Nevertheless, ion movement is limited at high frequencies, but the excellent mechanical properties provide an attractive advantage for flexible applications. To implement complementary integrated circuits with low power consumption, ambipolar SWCNT TFTs have attracted significant interest.^152^, ^159^ Xu et al. reported the polarity conversion method, which enables selectively converted n‐type TFTs by doping with diluted ethanolamine on p‐type SWCNTs.^151^ This strategy provided practical insights to the fabrication of printed CMOS inverters consisting of p‐type and n‐type SWCNT TFTs. However, even though many research groups have conducted intensive studies such as chemical doping, the electrical characteristics are typically inferior to those of n‐type SWCNTs due to their inherent instability in ambient conditions, and so, metal oxide TFTs can be regarded as a substitute n‐type counterpart for realizing high‐performance complementary logics exhibiting voltage inversion at half of the *V*_DD_.^125^ Recent efforts to implement printed complementary circuits including inverters^242^ and multistage ring‐oscillators^243^ on flexible substrates are summarized in **Table** **1**. As an important achievement, Grubb et al. focused on short‐channel inkjet‐printed SWCNT TFTs to enhance the operating speed.^158^ A short‐channel of 1 µm was formed by the different wetting properties between the nonaqueous silver ink and the aqueous silver ink, which allowed high intrinsic cutoff frequency (*F*_T_) of 18.21 GHz at a much lower gate capacitance of 0.093 pF. Recently, Cai et al. reported fully printed stretchable SWCNT TFTs with unsorted CNTs and high‐purity semiconducting SWCNTs (**Figure** **16**).^162^ By employing a hybrid gate dielectric layer composed of PDMS and barium titanate (BaTiO_3_), relatively high‐k dielectric was realized depending on the ratio of BaTiO_3_. In addition, inherently excellent mechanical stability and compatibility with the PDMS substrate of the fabricated TFTs were observed maintaining their electrical characteristics including mobility and on/off ratio even under a tensile strain of 50% for 1400 cycles without delamination. This result indicates that these are one of the best choices to realize practical stretchable TFT applications due to their low‐cost, scalability, and elastomeric substrate‐compatible low‐temperature process.

**Table 1:** Summary of recent progress on inkjet‐printed SWCNT field‐effect transistors

Ink material	Solvent	Substrate	*S*/*D* contact	Dielectric	Semiconductor	Key electrical characteristics of TFT	Application	Ref.
SWCNT	Toluene	HfO_2_/*n*‐doped Si, SiO_2_/Mo Glass	Ag	HfO_2_, SiO_2_	SWCNT	p‐type = 10–30 cm^2^ V^−1^ s^−1^ log(*I*_On_/*I*_Off_) = 6–7 n‐type = 10–30 cm^2^ V^−1^ s^−1^ lo*g*(*I*_On_/*I*_Off_) = 6	Inverter Noise margin = 103% @ 1/2 *V_dd_* Voltage gain = 30 Low static power consumption = 0.1 µW @ *V_dd_* = 1 V	151
SWCNT	Toluene (with P‐DPPb5T)	HfO_2_/*n*‐doped Si	Au	HfO_2_	SWCNT	= 33.2 cm^2^ V^−1^ s^−1^ log(*I*_On_/*I*_Off_) ≈ 7	Inverter Noise margin = 74 % @ 1/2 *V_dd_* Voltage gain = 16 @ 1 V	152
SWCNT	Toluene	PTS coated SiO_2_	Ti/Pd	SiO_2_	SWCNT	*w*/alignment ≈ 1 cm^2^ V^−1^ s^−1^ log(*I*_On_/*I*_Off_) ≈ 2–3 *w*/*o* alignment ≈ 0.1 cm^2^ V^−1^ s^−1^ log(*I*_On_/*I*_Off_) ≈ 5		156
SWCNT	1‐cyclohexyl‐2‐pyrrolidone	Glass	Ag	Al_2_O_3_	SWCNT	p‐type(acetone exposure) = 7.3 ± 1.8 cm^2^ V^−1^ s^−1^ log(*I*_On_/*I*_Off_) = 2.36 ± 0.25 n‐type(ambipolar) = 2.4 ± 0.4 cm^2^ V^−1^ s^−1^ log(*I*_On_ */I*_Off_) = 2.27± 0.23	Inverter Voltage gain = 12 Ring oscillator Frequency = 2.91 kHz	159
SWCNT or P3‐mSWCNT	Toluene (sSWCNT) DI(P3‐mSWCNT)	SiO_2_	Ag, Au, mSWCNT	SiO_2_	SWCNT	= 6.7 cm^2^ V^−1^ s^−1^ _intrinsic_ = 3.4 cm^2^ V^−1^ s^−1^ log(*I*_On_ */I*_Off_) ≈ 6	Electrode *R_c_* = 16.8 kΩ µm *R_s_* = 390 Ω/sq	160
SWCNT or P3‐SWCNT	DI water with Triton X‐100	Polydimethylsiloxane (PDMS)	P3‐SWCNT	High‐k barium titanate nanoparticles / PDMS	SWCNT	= 7 cm^2^ V^−1^ s^−1^ log(*I*_On_ */I*_Off_) ≈ 3–4	Electrode *R_s_* > 100 kΩ Inverter Voltage gain > 0.5	162
sSWCNT	99% SWCNT solution (NanoIntegris, Inc.)	PET	Ag	High‐k barium titanate nanoparticles / poly(methyl methacrylate)	sSWCNT	= 4.27 ± 1.62 cm^2^ V^−1^ s^−1^ log(*I*_On_ */I*_Off_) = 4.55 ± 0.87		241

**Figure 17:**
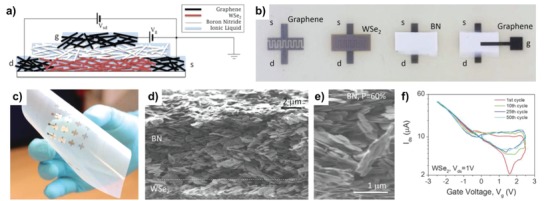
All‐printed, all‐nanosheet TFT. a) Schematic showing all‐printed TFT structure. The source, drain, and gate electrodes are inkjet‐printed networks of graphene nanosheets; the channel is an inkjet‐printed network of WSe_2_ nanosheets. The gate electrode is separated from the channel by a spray‐cast BN nanosheet network. The entire porous volume of the structure is filled with an ionic liquid to facilitate electrolytic gating. b) Photographs of the printing steps. From left to right: Graphene source (s) and drain (d) electrode (*t* ≈ 400 nm); the WSe_2_ channel (*t* ≈ 1 mm, *L* = 200 mm, *w* = 16 mm); the BN separator (*t* ≈ 8 mm); and finally the graphene gate (*g*, *t* ≈ 400 nm). c) A flexible array of printed TFTs. d) Cross‐sectional SEM image showing WSe_2_ channel and BN separator. e) Magnified image of BN network showing porosity (*P* = 60%). f) Transfer curves for a printed TFT with a WSe_2_ active channel after cycling the gate voltage 1, 10, 25, and 50 times. Reproduced with permission.^179^ Copyright 2017, American Association for the Advancement of Science.

**Figure 18:**
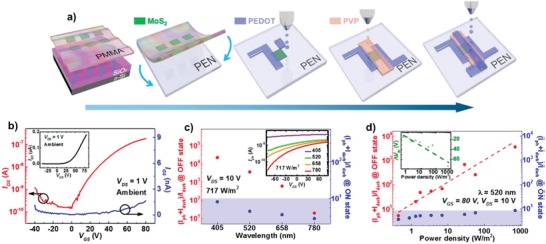
a) Schematic illustration of the fabrication processes for fully printed, flexible, and transparent CVD‐synthesized MoS_2_ phototransistors. b) *I*_DS_–*V*_GS_ curves using a log scale at *V*_DS_ = 1 V. Inset represents the *I*_DS_−*V*_GS_ curves using a linear scale. The ratio of *I*_light_ (= *I*_ph_ + *I*_dark_) to *I*_dark_ in the ON and OFF states as a function of c) wavelength and d) laser power at a fixed *V*_DS_ = 10 V. Insets of (c) and (d) exhibit *I*_ph_ versus *V*_GS_ and the change in *V*_th_ with respect to the laser power, respectively. As the laser power increased, *V*_th_ shifted in the negative voltage direction, which indicates an increase in *I*_ph_ in the subthreshold regime. Reproduced with permission.^247^ Copyright 2017, American Chemical Society.

Regarding inkjet‐printed TFTs with 2D semiconductors, many efforts have been reported to widen the bandgap of graphene by doping boron or organic molecules on the surface. Additionally, nonzero bandgap TMDCs are widely pursued for realizing 2D semiconductor TFT applications as summarized in **Table** **2**. As a promising strategy for large‐area printed productions, liquid‐phase exfoliation has been widely used to make 2D inks. Torrisi et al. reported inkjet‐printed graphene TFTs with liquid‐phase exfoliated graphene semiconducting layers and electrodes.^176^ Although relatively poor switching characteristics, exhibiting an on/off ratio of 10 corresponding large SS values were observed, this result showed promising potential for inkjet‐printed transparent 2D electronics with an excellent transparency of 80%. Since then, liquid‐phase exfoliated graphene has widely been employed to deposit conductive layers using a variety of methods, such as rod coating, inkjet printing, and gravure printing, to realize flexible and transparent printed electronics.^57^, ^244^, ^245^ As insulating separators, *h*‐BN inks dispersed in IPA or DMF were deposited using the spray‐coating and screen‐printing methods to realize vertically stacked heterostructures,^180^, ^181^ but still many issues remain to be overcome for realizing fully printed TFTs with 2D materials. In 2017, all‐printed TFTs with liquid‐phase exfoliated 2D nanosheets on a flexible substrate were finally reported (**Figure** **17**).^179^ The vertically stacked TFTs consisted of graphene drain, source, and gate electrodes; a BN dielectric, and various TMDCs, such as MoS_2_, MoSe_2_, WS_2_, and WSe_2_ dispersed in NMP. In addition, to enhance the switching operation by the BN network, an ionic liquid was introduced, allowing electrolytic gating. Although their electrical performance including a carrier mobility over 0.1 cm^2^ V^−1^ s^−1^ and an on/off ratio over 10^2^ was inferior to that of FETs with mechanically exfoliated TMDCs, this important achievement indicated the possibility to utilize various 2D semiconducting inks for realizing printed 2D electronics. In another strategy, large‐area 2D TFT arrays were reported with transfer‐printed 2D MoS_2_ and inkjet‐printed components. Because conventional electron‐beam nanolithography or photolithography techniques to define electrodes and insulating layers on atomically thin 2D channels are not compatible with large‐area flexible platforms, Kim et al. reported the fabrication of large‐area CVD‐grown MoS_2_ FETs with inkjet‐printed silver contacts,^246^ and then, as a following work, they inkjet‐printed conductive polymer electrodes and cross‐linked polymer dielectric layers directly onto the transfer‐printed MoS_2_ channel layer in ambient conditions.^247^ They fabricated highly transparent top‐gated phototransistors exhibiting electronically tunable photoswitching properties including a photoresponsivity of ≈0.1 W A^−1^ and an external quantum efficiency of ≈8%, which are comparable to those of MoS_2_ phototransistors fabricated by conventional deposition methods. These approaches also provided key insights to implement large‐area, low‐cost, transparent, and flexible 2D electronics (**Figure** **18**).

**Table 2:** Summary of recent progress on inkjet‐printed 2D field‐effect transistors and capacitors

Ink material	Solvent	Substrate	*S*/*D* contact	Dielectric	Semiconductor	Key electrical characteristics	Application	Ref
rGO	20% ethylene glycol and 0.5g L‐^1^ sDBS	PET	rGO	Poly(methyl methacrylate)	GO	= 8 cm^2^ V^−1^ s^−1^ log(*I*_On_/*I*_Off_) = 4 *R*_s_ = 25 kΩ sq^−1^	Field‐effect transistors (FETs)	177
rGO	Ascorbic acid VC	PET, PDMS, SiO_2_/Si	IrGO	SiO_2_	GO	_e_ = 4.37 cm^2^ V^−1^ s^−1^ _h_ = 0.68 cm^2^ V^−1^ s^−1^ *R*_s_ = 0.6 kΩ sq^−1^	FETs	178
MoS_2_	N‐Methyl‐2‐pyrrolidone	PET	Graphene	*h*‐BN	MoS_2_	= 0.15 cm^2^ V^−1^ s^−1^ log(*I*_On_/*I*_Off_) ≈ 2	FETs	179
MoSe_2_	N‐Methyl‐2‐pyrrolidone	PET	Graphene	*h*‐BN	MoSe_2_	= 0.18 cm^2^ V^−1^ s^−1^ log(*I*_On_/*I*_Off_) ≈ 2	FETs	179
WS_2_	N‐Methyl‐2‐pyrrolidone	PET	Graphene	*h*‐BN	WS_2_	= 0.22 cm^2^ V^−1^ s^−1^ log(*I*_On_/*I*_Off_) ≈ 2–3	FETs	179
WSe_2_	N‐Methyl‐2‐pyrrolidone	PET	Graphene	*h*‐BN	WSe_2_	= 0.08 cm^2^ V^−1^ s^−1^ log(*I*_On_/*I*_Off_) ≈ 2	FETs	179
*h*‐BN	Isopropyl alcohol	alumina‐coated PET	Graphene	*h*‐BN	None	Unit capacitance = 0.24–1.1 nF cm^−2^ *R* = 120 kΩ	Capacitor	180
*h*‐BN	Dimethylformamide	BoPET	Cu, Ag	*h*‐BN	None	ε ≈ 2.57 F/m@1 MHz	Flexible capacitor	181
Graphene	Dimethylformamide, exchanged to terpineol	Si	Ag	SiO_2_	Graphene	≈ 0.12 cm^2^ V^−1^ s^−1^ log(*I*_On_/*I*_Off_) ≈ 0.1	FET Supercapacitor *C*_specific_ = 0.59 mF cm^−2^ Response time = 13 ms	245

### 3.4 Micro‐Electromechanical Relays

**Figure 19:**
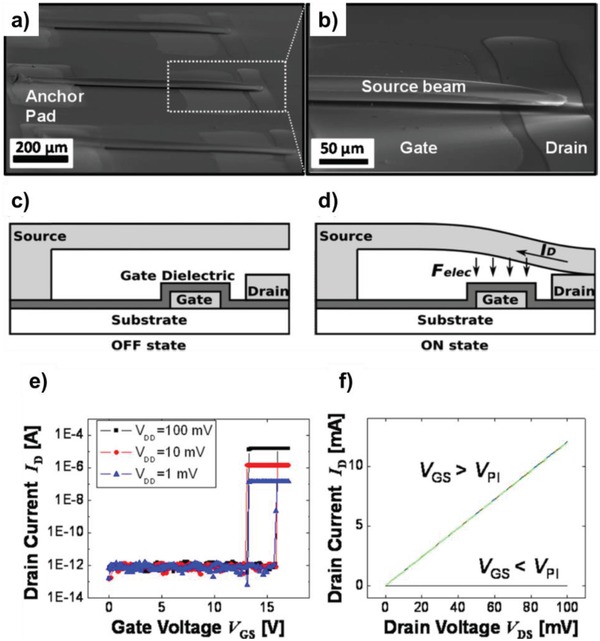
Printed MEM switch fabricated using nanoparticle ink. Scanning electron micrographs of a) multiple printed MEM switches and b) close‐up view of one switch. The source beam is anchored to the source pad; the two other electrodes (actuating gate and contacting drain) are located underneath the source beam. Schematic cross‐sectional illustration of the three‐terminal switch structure c) in the OFF‐state and d) in the ON‐state. Measured e) *I*_DS_–*V*_GS_ and f) *I*_DS_–*V*_DS_ characteristics of the printed MEM switch. Reproduced with permission.^252^ Copyright 2013, American Chemical Society.

**Figure 20:**
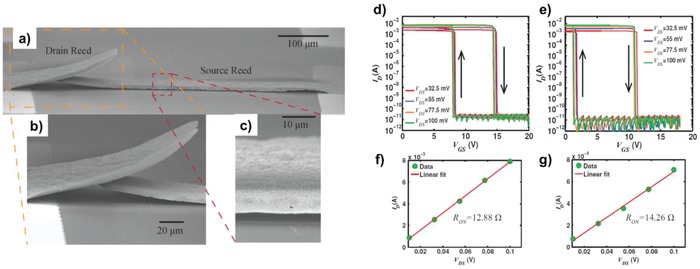
SEM images of inkjet‐printed MEM reed relay. a) Drain reed blocks the curling of source reed, b) close‐up image of the drain and the source reed contact region, c) close‐up image of the suspended source reed showing the air gap. Switching characteristics of inkjet‐printed reed relays. *I*_DS_–*V*_GS_ characteristics of the reed relay with varying drain bias (*V*_DS_) for d) 5.5 µm and e) 4.5 µm thick source reed showing abrupt switching with turn‐off voltages (*V*_TOF_) of ≈15 and ≈11 V, respectively. These devices also show high on‐current and noise level off‐current that are in the noise floor of the parameter analyzer. Very low on resistances (*R*_ON_) of 12.88 and 14.26 Ω are extracted from the *I*_DS_–*V*_DS_ characteristics for f) 5.5 µm and g) 4.5 µm thick source reed, respectively. Reproduced with permission.^255^ Copyright 2015, Wiley‐VCH.

Due to tremendous achievements in terms of electronics materials, device structures, and patterning strategies, printed TFTs have shown drastically improved electrical characteristics in terms of carrier mobility and switching speed. However, further enhancements are still needed in their power consumption, environmental stability, SS, and on/off ratio for a wide range of applications, especially display or solar applications, which require perfectly low off‐state currents. An alternative approach that is particularly attractive for applications requiring ultralow device leakage is the use of printed MEM relays with movable cantilevers operated by electrostatic actuation.^247^, ^248^, ^249^, ^250^, ^251^ Because a semiconductor layer is not required in mechanical switching devices with an extremely low on‐state resistance, off‐state leakage at the noise level, and an SS value below 1 mV, MEM relays are candidates for use in both pixel switches and high power switching electronics, such as analog shift registers. The absence of semiconductors in the channel layers of MEM relays also imparts excellent environmental stability without temperature dependency for the carrier‐transport or carrier‐injection properties which should be considered in the field of FETs. Although such mechanical switches have received much attention due to these attractive advantages, only a few papers have been reported for inkjet‐printed MEM relays due to the difficulty in fabricating 3D cantilevers with sufficient strength to be suspended. In addition, undesirable stiction between the conductive channel and counterpart electrodes should be overcome to minimize hysteresis of the switching properties. Finally, the relatively high operating voltage is also a critical bottleneck to various electronics applications. In this section, we review the state of the art of inkjet‐printed MEM relays and their applications focusing on novel approaches to address these issues. Park et al. reported, for the first time, three‐terminal inkjet‐printed MEM relays, such as three‐terminal transistors (**Figure** **19**).^252^ On the inkjet‐printed *S*/*D* electrodes, cross‐linked poly(4‐vinylphenol) (PVP) and PMMA layers, which worked as the gate dielectric and sacrificial layers, respectively, were sequentially deposited by spin coating to obtain a uniform film thickness. To minimize the hysteresis caused by the stiction force between the cantilever and drain electrode, a few Ag ink droplets were placed on the contact region on the drain electrode to introduce the coffee‐ring effect. This approach is an efficient method to reduce the contact area, which is a critical factor in determining the stiction force. On the sacrificial layer, Ag cantilevers with a thickness of 2.2 µm were inkjet‐printed in 5‐pass to fabricate more strongly suspended cantilevers. Then, a via‐hole was formed at the end of the cantilever to connect the cantilever and underlying source electrode by etching the sacrificial layer weakened by exposure to UVO. After filling the via‐hole with printed Ag ink, the PMMA sacrificial layer was removed by dipping in acetone. This procedure gave the inkjet‐printed Ag cantilever the freedom to move downward upon application of a gate bias due to the air gap between the gate and drain electrodes. The suspended cantilever could mechanically touch the drain electrode upon application of a gate bias of 16.7 V and be released by reducing the gate bias to below 13 V. The fabricated MEM relays show ideal operations including abrupt switching, extremely low contact resistance between metal electrodes, and a high on/off ratio over 10^8^. These results showed the promising possibility to realize inkjet‐printed MEM relays by introducing multipass printing and the coffee‐ring effect on the channel cantilever and underlying drain electrode, respectively. However, further studies to deliver mechanically higher stiffness on the suspended cantilever and reduce the operation voltage were highly desired. In 2014, Chung et al. reported a new approach to realize strongly suspended cantilevers by realizing an enhanced moment of inertia of the cantilever, which directly affects its stiffness by exploiting the coffee‐ring effect.^253^ By optimizing the solvent combination, substrate temperature during cantilever printing, and intermediate drying conditions, the coffee‐ring effect could be fully introduced or eliminated during cantilever printing. The coffee‐ring effect formed high ridges along the cantilever which dramatically enhanced the mechanical stiffness over 100 times with printing only 5 passes compared to that of the cantilever without exploiting the coffee‐ring effect. This strategy is feasible only in inkjet printing. The number of printing passes, which is closely related to the stiffness of the cantilever, and the air gap were also carefully optimized resulting in a much improved device‐yield with a reduced operating voltage of 6.6 V and a hysteresis window below 2 V. In addition, introducing a double‐clamped beam and ridges on the drain by the coffee‐ring effect are also promising approaches to fabricate a strongly suspended beam and reduce the stiction force to result in less hysteresis, respectively.^250^ With these efforts, inkjet‐printed four‐terminal MEM relays and an inverter enabled CMOS‐like operation with the associated switching behavior relative to a body electrode with a sharp transition in both directions. The implementation of a four‐terminal device is critically important, because it allows for the realization of large‐area and energy‐effective fully complementary logic functions.^254^ Because the optimization of the mechanical stiffness of a suspended cantilevers and stress variation of inkjet‐printed films is difficult, inkjet‐printed three‐terminal MEM reed relays were demonstrated to offer excellent tolerance to mechanical stress variations that occur across the cantilever during the annealing process.^255^, ^256^ By employing a relatively short blocking reed, the upward curling of the channel reed due to the stress gradient in the film enabled physical contact between the two reeds, thus delivering immunity to stress variations (**Figure** **20**). Most recently, inkjet‐printed MEM relays were employed as substitutes for conventional solar bypass applications. Due to the extremely low on‐resistance below 5 Ω and high current carrying capabilities over 100 mA, such MEM relays could be an attractive switching system for rerouting solar cell arrays in the shade.^257^

In terms of further improvements, optimization of the geometrical parameters to deliver appropriate mechanical stiffness and electrostatic scaling of the mechanical switches, especially for low static‐power and dynamic‐power applications, is highly desirable. Although dimensional scaling is still challenging, advantages that are feasible only in inkjet printing, for example, exploitation of the coffee ring effect, offer promising solutions to realizing large‐area and environmentally insensitive switching devices or actuators.

## 4 Conclusions and Future Outlook

**Figure 21:**
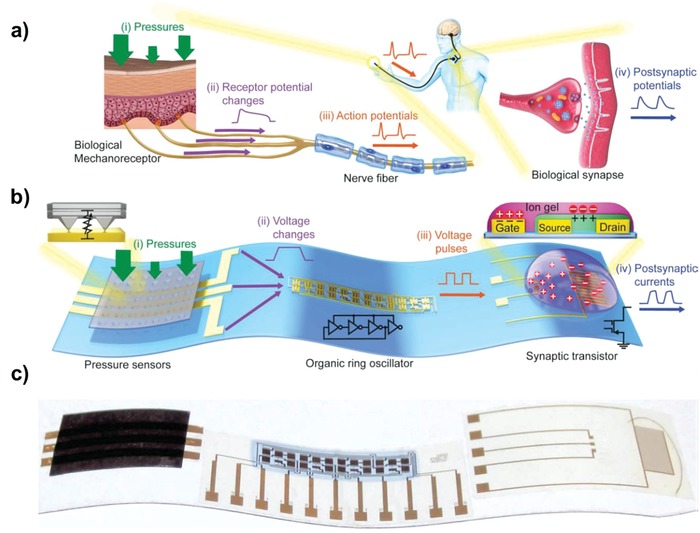
An artificial afferent nerve system in comparison with a biological one. a) A biological afferent nerve that is stimulated by pressure. Pressures applied onto mechanoreceptors change the receptor potential of each mechanoreceptor. The receptor potentials combine and initiate action potentials at the heminode. The nerve fiber forms synapses with interneurons in the spinal cord. Action potentials from multiple nerve fibers combine through synapses and contribute to information processing. b) An artificial afferent nerve made of pressure sensors, an organic ring oscillator, and a synaptic transistor. Only one ring oscillator connected to a synaptic transistor is shown here for simplicity. However, multiple ring oscillators with clusters of pressure sensors can be connected to one synaptic transistor. The parts with the same colors in (a) and (b) correspond to each other. c) A photograph of an artificial afferent nerve system. Reproduced with permission.^261^ Copyright 2018, American Association for the Advancement of Science.

**Figure 22:**
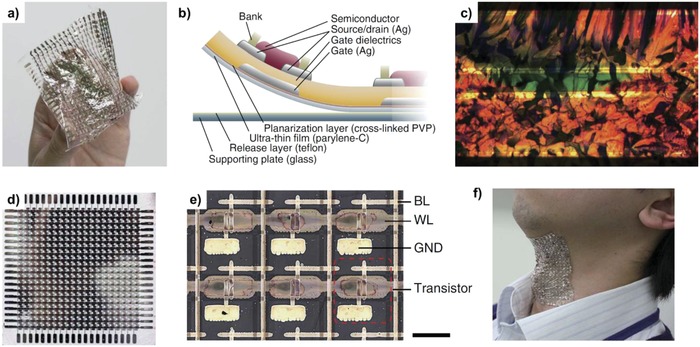
Fully printed organic thin‐film transistors on ultraflexible films. a) A photograph of organic TFT devices on 1 mm thick parylene‐C films. The devices were fabricated entirely with printing processes. Scale bar, 2 cm. b) Cross‐sectional diagram of a thin organic TFT device. c) A polarization microscope image of the channel region. Scale bar, 100 mm. d) Top‐view photograph of a completed 10 cm × 10 cm fully printed 20 × 20 TFT array fabricated on an ultraflexible parylene‐C film. Scale bar, 1 cm. e) A magnified view of six TFT devices. Scale bar 2 mm. f) Flexible TFT array sheet conforming to a human throat. Reproduced with permission.^196^ Copyright 2014, Springer Nature.

For the last two decades, inkjet printing has gained considerable attention as a promising process for manufacturing low‐cost, low‐temperature, and large‐area electronics, therefore, representing a solid foundation in the field of flexible TFTs. A primary advantage that is feasible only in inkjet printing is the mask‐less patterning with a high degree of freedom. This ability allows easy customization by implementing a variety of complex electronic components, such as driving TFTs, memory devices, displays, sensors, and power supplies, on the same substrate using additive manufacturing. Additionally, inkjet printing must inherently employ low‐viscosity inks, which affords an opportunity to utilize the liquid dynamics before drying or sintering. The most well‐known example is selective patterning from different wetting properties for large‐area patterning.^258^, ^259^ Furthermore, strategies with the short‐channel formation with SAM‐treated *S*/*D* electrodes,^209^ by capillary forces,^260^ single‐crystal organic semiconductor formation by double‐shot printing,^100^ and channel self‐defined by the height of the *S*/*D* electrodes^188^ are facilitated only in inkjet printing and not available for printing employing high‐viscosity inks. In the most recently reported result, the coffee‐ring effect, which many studies have attempted to suppress to obtain uniformly deposited patterns, was fully exploited to enhance the mechanical stiffness by forming high ridges along the printed patterns.^253^, ^254^ Consequently, inkjet printing is not only a patterning method in the recent progress, but also a uniquely feasible approach to exploit the liquid dynamics before ink solidification. The strength of inkjet printing also lies in the realization of TFTs with vertically stacked structures thanks to a noncontact additive manufacturing ability. To achieve further improved electrical characteristics, high‐quality electronic inks with optimized rheological characteristics have been developed as along with inkjet printing and post‐thermal treatments. Specifically, low‐temperature oxide and emerging 2D inks with optimized surface tension, viscosity, and concentration have been intensively investigated for the realization of flexible and transparent TFTs. In addition, low‐temperature postannealing techniques that are compatible with flexible platforms have been developed. In these regards, the recently reported results have certainly shown the promise of inkjet printing for realizing next‐generation electronics, such as stretchable and transparent electronics on large‐area wearable platforms. Despite these efforts, however for the industrialization of inkjet‐printed TFTs beyond laboratory‐scale applications, many challenges still remain in terms of electrical performance and device‐yield. Although individual printable functional materials including conductive, insulating, and semiconductor inks have shown sufficient electrical performance to lead printing technologies toward the commercial phase, significant breakthrough is still needed to implement high yield multifunctional electronic systems, such as bioinspired artificial neural systems (**Figure** **21**).^261^ In addition, because its relatively poor resolution of inkjet printing is still one of the critical bottlenecks to industrialization, inkjet printing is not suitable for fabricating highly integrated systems that can be manufactured by high‐cost silicon fabrication. To address this issue, more reliable inkjet process should be developed from sub‐micrometer scale nozzles fabricated by using matured silicon processing. Also, more stable electronic inks with the consideration of their rheology should be investigated to enter the industrial phase. However, in the positive perspective, inkjet printing is indubitably a promising processing to provide great freedom in terms of the target platform, design rule, and mass customization by fully taking advantage of printing processes (**Figure** **22**).^196^ Furthermore, easily accessable inkjet printing is competitive to the realization of deposable electronics, such as wearable medical applications, low‐cost RFID tags, RF sensors, food packaging, and potentially flexible memory applications.^262^, ^263^, ^264^, ^265^, ^266^ Therefore, we believe that if these issues can be addressed, inkjet printing will provide a promising pathway for realizing low‐cost, lightweight, and easily customized wearable thin‐film electronics connected via the IoT.

## Conflict of Interest

The authors declare no conflict of interest.
